# Suicide Deaths Before and During the COVID 19 Pandemic

**DOI:** 10.1192/j.eurpsy.2023.267

**Published:** 2023-07-19

**Authors:** R. Rossom, R. Penfold, A. Owen-Smith, G. Simon, B. Ahmedani

**Affiliations:** 1 HealthPartners Institute; 2 University of Minnesota, Minneapolis; 3 Kaiser Permanente Washington, Seattle; 4 Georgia State University, Atlanta, United States; 5 Kaiser Permanente Washington, Seattle; 6 Henry Ford Health, Detroit

## Abstract

**Introduction:**

With stressors that are often associated with suicide increasing during the coronavirus disease 2019 (COVID-19) pandemic, there has been concern that suicide mortality rates may also be increasing. Our objective was to determine whether suicide mortality rates increased during the COVID-19 pandemic.

With stressors that are often associated with suicideincreasing during the coronavirus disease 2019 (COVID-19) pandemic,there has been concern that suicide mortality rates may alsobe increasing.

**Objectives:**

Our objective was to determine whether suicidemortality rates increased during the COVID-19 pandemic.

**Methods:**

We conducted an interrupted time-series study using data from January 2019 through December 2020 from 2 large integrated health care systems. The population at risk included all patients or individuals enrolled in a health plan at HealthPartners in Minnesota or Henry Ford Health in Michigan. The primary outcome was change in suicide mortality rates, expressed as annualized crude rates of suicide death per 100,000 people in 10 months following the start of the pandemic in March 2020 compared with the 14 months prior. We conducted an interrupted time-series study using data fromJanuary 2019 through December 2020 from 2 large integrated health care systems. The population at risk included all patients or individuals enrolledin a health plan at HealthPartners in Minnesota or Henry Ford HealthSystem in Michigan. The primary outcome was change in suicide mortality rates, expressed as annualized crude rates of suicide death per 100,000 people in 10 months following the start of the pandemic in March2020 compared with the 14 months prior.

**Results:**

There were 6,434,675 people at risk in the sample, with 55% women and a diverse sample across ages, race/ethnicity, and insurance type. From January 2019 through February 2020, there was a slow increase in the suicide mortality rate, with rates then decreasing by 0.45 per 100,000 people per month from March 2020 through December 2020 (SE= 0.19, P=0.03). There were 6,434,675 people at risk in the sample, with 55% women and a diverse sample across ages, race/ethnicity, and insurance type. From January 2019 through February 2020, there was a slow increase in the suicide mortality rate, with rates then decreasing by 0.45 per 100,000 people per month from March 2020 through December 2020 (SE= 0.19, P=0.03).

**Conclusions:**

Overall suicide mortality rates did not increase with the pandemic, and in fact slightly declined from March to December 2020. Our findings should be confirmed across other settings and, when available, using final adjudicated state mortality data. Overall suicide mortality rates did not increase with the pandemic, and in fact slightly declined from March to December 2020. Our findings should be confirmed across other settings and,when available, using final adjudicated state mortality data.

**Disclosure of Interest:**

None Declared

